# Supplementary material for: Causal Relationship between Aging and Anorexia Nervosa: A White-Matter-Microstructure-Mediated Mendelian Randomization Analysis
Source: Biomedicines. 2024 Aug 16;12(8):1874. doi: 10.3390/biomedicines12081874 (PMC11351342; doi:10.3390/biomedicines12081874)
Supplement: Supplementary file 1 [file biomedicines-12-01874-s001.zip › Supplementary Figure/Figure S7/Single SNP plot of MR analysis for ubm-b-1886 to AN.pdf]

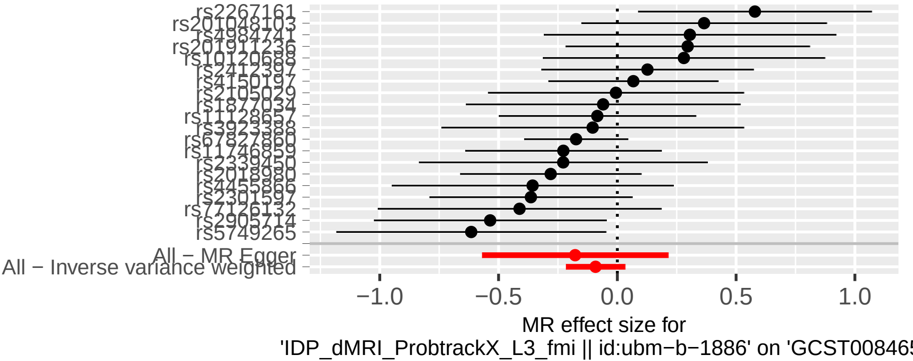

rs2267161
rs201048103
rs4984741
rs201911236
rs10120688
rs2412397
rs4150197
rs2105029
rs1877034
rs11128657
rs3923388
rs67827860
rs11746859
rs2339450
rs2018980
rs4455866
rs2301597
rs77126132
rs2905714
rs5749265
All − MR Egger
All − Inverse variance weighted
−1.0
−0.5
0.0
0.5
1.0
MR effect size for
'IDP_dMRI_ProbtrackX_L3_fmi || id:ubm−b−1886' on 'GCST00846
